# Role of Brain Arterial Remodeling in HIV-Associated Cerebrovascular Outcomes

**DOI:** 10.3389/fneur.2021.593605

**Published:** 2021-06-22

**Authors:** Antonio Spagnolo-Allende, Jose Gutierrez

**Affiliations:** Department of Neurology, Columbia University Irving Medical Center, New York, NY, United States

**Keywords:** HIV, brain, arterial remodeling, cerebrovascular disease, HIV-associated neurocognitive impairment

## Abstract

As the life expectancy of people living with HIV (PLWH) on combination antiretroviral therapy (cART) increases, so does morbidity from cerebrovascular disease and neurocognitive disorders. Brain arterial remodeling stands out as a novel investigational target to understand the role of HIV in cerebrovascular and neurocognitive outcomes. We therefore conducted a review of publications in PubMed, EMBASE, Web of Science and Wiley Online Library, from inception to April 2021. We included search terms such as HIV, cART, brain, neuroimmunity, arterial remodeling, cerebrovascular disease, and neurocognitive disorders. The literature shows that, in the post-cART era, PLWH continue to experience an increased risk of stroke and neurocognitive disorders (albeit milder forms) compared to uninfected populations. PLWH who are immunosuppressed have a higher proportion of hemorrhagic strokes and strokes caused by opportunistic infection and HIV vasculopathy, while PLWH on long-term cART have higher rates of ischemic strokes, compared to HIV-seronegative controls. Brain large artery atherosclerosis in PLWH is associated with lower CD4 nadir and higher CD4 count during the stroke event. HIV vasculopathy, a form of non-atherosclerotic outward remodeling, on the other hand, is associated with protracted immunosuppression. HIV vasculopathy was also linked to a thinner media layer and increased adventitial macrophages, suggestive of non-atherosclerotic degeneration of the brain arterial wall in the setting of chronic central nervous system inflammation. Cerebrovascular architecture seems to be differentially affected by HIV infection in successfully treated versus immunosuppressed PLWH. Brain large artery atherosclerosis is prevalent even with long-term immune reconstitution post-cART. HIV-associated changes in brain arterial walls may also relate to higher rates of HIV-associated neurocognitive disorders, although milder forms are more prevalent in the post-cART era. The underlying mechanisms of HIV-associated pathological arterial remodeling remain poorly understood, but a role has been proposed for chronic HIV-associated inflammation with increased burden on the vasculature. Neuroimaging may come to play a role in assessing brain arterial remodeling and stratifying cerebrovascular risk, but the data remains inconclusive. An improved understanding of the different phenotypes of brain arterial remodeling associated with HIV may reveal opportunities to reduce rates of cerebrovascular disease in the aging population of PLWH on cART.

## Introduction

Despite the effectiveness of modern combination antiretroviral therapy (cART), HIV infection continues to be frequently accompanied by cerebrovascular disease and cognitive decline (1–3). Arterial remodeling is emerging as a possible link between HIV infection and cerebrovascular disease and, possibly, cognitive disorders (4–6). However, our understanding of the pathogenic role of HIV in arterial remodeling, especially among the increasing population of people living with HIV (PLWH) in long-term cART, remains limited. In this review, we will discuss potential mechanisms underlying HIV-associated arterial remodeling. We will also summarize our current understanding of the potential role that HIV-associated arterial remodeling plays in cerebrovascular disease and cognitive disorders among PLWH, especially among those aging with HIV and in cART. To that end, we conducted a review of publications in PubMed, EMBASE, Web of Science, and Wiley Online Library, from inception to April 2021. We included search terms such as HIV, cART, brain, neuroimmunity, arterial remodeling, cerebrovascular disease, stroke, and neurocognitive disorders.

## HIV and Cerebrovascular Disease in the cART ERA

The life expectancy of PLWH is now generally approaching that of the general HIV-seronegative population, thanks to the advent of cART in the mid-1990s (7–9). Although fewer PLWH are dying of AIDS-related diseases, the prevalence of comorbidities in PLWH that are not caused by AIDS, such as cardiovascular disease, remains high compared with HIV-seronegative controls. Cardiovascular disease is the second leading, non-AIDS cause of death among PLWH in the United States, and third in Europe (10). This includes cerebrovascular disease, which remains prevalent among PLWH even after the widespread adoption of cART (11–13). For instance, while the seminal Strategic Timing of Antiretroviral Therapy (START) study demonstrated a 40% reduction in AIDS-related diseases with early cART administration (14), early cART and lower AIDS rates did not preclude increased cerebrovascular risk in PLWH (15).

Studies conducted in high-income countries have established that PLWH in the post-cART era have a 1 to 5% population burden of stroke, while 4 to 34% show ischemic brain lesions on autopsy (16–19). In the United States (US), the total number of primary stroke diagnoses made in PLWH rose by 67% between 1997 and 2006, according to a population study of hospital stroke diagnoses (13). The timing of this increase, quite notably, coincided with the propagating adoption of cART and the simultaneous decrease, by 7%, of stroke admissions in the general population. The authors remarked that the case increase was mostly caused by a rise in ischemic rather than hemorrhagic stroke hospitalizations among PLWH. They also reported that the proportion of PLWH hospitalized for ischemic stroke had more than doubled in the studied period. In a 2019 analysis of a large US healthcare claims database (20), stroke rates in PLWH were shown to be nearly triple that of HIV-seronegative controls, adjusting for sex and age. In 2015, men living with HIV enrolled in the US-based Veterans Aging Cohort Study were still shown to have an increased risk of ischemic stroke compared to HIV-seronegative controls (21). Hemorrhagic stroke, on the other hand, was more frequently seen in the immunosuppressed than those on stable cART (22, 23). In Europe, a Danish population-based cohort study showed the incidence of cerebrovascular events in PLWH was 1.6 times that of HIV-seronegative controls, adjusting for traditional vascular risk factors (24). This rate, it should be noted, may indeed be higher. Since both HIV and cART are independently associated with traditional cardiovascular risk factors, adjusting for them may have negatively biased those results (25–28). Stroke burden among PLWH in low-to-middle-income countries is less well-defined, but a higher burden of HIV and AIDS, along with increasing prevalence of traditional risk factors, makes these populations even more vulnerable to cardiovascular events (29, 30). In Malawi, for instance, the second most common cause of stroke in 2017 was HIV (29) and in 2010, a reference hospital in Tanzania reported that 20% of stroke cases co-occurred with HIV infection (31).

The post-cART rise in HIV-associated stroke events, some researchers originally suspected, could be caused by the overall increase in HIV infections, possible side effects of new cART drugs, improved survival on cART, or even just better recognition of stroke symptoms among PLWH (2). The exact causes, however, remain difficult to pin down even today. Studies trying to determine stroke rates in HIV-positive populations often rely on incomplete data regarding cardiovascular risk factors and on standardized disease codes that may not appropriately capture cerebrovascular events among PLWH (12). Nonetheless, the literature strongly supports the existence of cerebrovascular risk in HIV infection beyond what would be explained by traditional risk factors. Compared to HIV-seronegative controls, PLWH with cerebrovascular disease may be younger and less likely to have the rates of high blood pressure and elevated cholesterol typically seen in stroke patients (11, 32, 33). Surveys of PLWH have shown a premature occurrence of stroke events compared to their HIV-seronegative counterparts, especially in low-to-middle-income countries (34).

Interestingly, a cohort of elite controllers (PLWH who maintain undetectable viral load without cART) still showed increased coronary atherosclerosis and biomarkers of immune activation compared to HIV-seronegative controls (35). This points to reasons other than high viremia and cART side effects to explain HIV-associated atherosclerosis. The SMART study also showed that interrupting cART in immunocompetent PLWH led to increase in cardiovascular events, compared to those whose cART continued after immune reconstitution, providing further support for the role of cART in prevention, rather than promotion, of cardiovascular disease in PLWH (36). A separate cohort of elite controllers showed higher median carotid intima-media thickness than seronegative controls, adjusting for traditional cardiovascular risk factors (37). Moreover, autopsy studies have found altered vascular caliber in the brain of PLWH who experienced long-term viral suppression before death (1, 5, 38–40).

A multicenter cohort study in the US also showed that the Framingham Stroke Risk Score underestimated long-term risk of stroke among men living with HIV (41). In fact, standard cardiovascular risk prediction functions that were developed for use in the general population tend to systematically underestimate risk in PLWH (26). This exposes the limitations in our current understanding of the pathophysiology of cerebrovascular disease among PLWH, which may differ from that of people who are HIV-seronegative. Even in the absence of AIDS, the inflammatory effects of HIV may be contributing to vascular disease in the brain, with varying effects depending on immunological and cART status (42). Inflammation-mediated vascular remodeling may therefore be playing a key role in HIV-associated cerebrovascular disease, beyond the isolated effects of chronically high viremia, which is much less commonly seen post-cART. Furthermore, the effects of cerebrovascular changes in HIV infection may not be limited to stroke: they may also have a role to play in the HIV-associated neurocognitive disorders, still prevalent in the post-cART era (43–46).

## HIV-Associated Endothelial Dysfunction

Arterial remodeling is the process in which arteries undergo structural and functional changes as a response to biological stimuli. A response that results in increase in arterial size is defined as outward remodeling (e.g., dolichoectasia, aneurysm), whereas a decrease in size or caliber is defined as inward remodeling (e.g., atherosclerosis) (47). Arterial remodeling can also be described as hypertrophic (thickening of the vascular wall), eutrophic (constant wall thickness) or hypotrophic (thinning of the wall) (48). When the endothelium is functionally intact, it senses stimuli such as blood flow and shear stress to modulate arterial remodeling (49, 50). A dysfunctional endothelium could alter the production or passage of vasoconstricting and vasodilating signals, thereby altering the natural course of arterial remodeling. Studies have shown that in PLWH, even after long-term cART, HIV-associated endothelial dysfunction can be present and flow-mediated dilation of arteries impaired (51–53). Endothelial dysfunction leads to abnormal clotting and increased nitric oxide production, altering vessel tone, permeability and chemokine expression. This leads to leukocyte transendothelial migration (2, 36, 54–56). The ultimate consequence of these changes is wall remodeling, plaque formation and/or increased presence of inflammatory cells in the vessel wall. As a consequence of HIV-induced arterial remodeling, both thrombus formation (cause of ischemic strokes) and possibly rupture (cause of hemorrhagic strokes) may be precipitated (2, 57).

While HIV-associated endothelial dysfunction (and its compromise of brain vasculature) is becoming a clearer independent entity in the literature, the mechanisms through which it may lead to arterial remodeling and adverse cerebrovascular outcomes remains unclear. HIV-1 is not understood to be directly vasculotropic. Endothelial cells do, however, express the receptors needed for viral entry (CD4 and CXCR5) (58). While viral replication does not take place in these cells, endothelial function may nonetheless be altered in ways that could initiate and propagate atherogenesis (2). Circulating HIV-infected immune cells, freely circulating HIV, HIV proteins (released by host cell lysis or actively secreted), and HIV-induced proinflammatory cytokines; all have the potential to activate the endothelium (59, 60). A 2011 biomarker study of 44 PLWH and 29 seronegative controls proposed soluble CD163 (sCD163), a monocyte- and macrophage-specific molecule, as a marker of HIV activity (61). In that study, in PLWH who initiated cART in early HIV infection (≤1 year), sCD163 decreased to levels comparable to HIV-seronegative individuals. In those who initiated cART later (>1 year after infection), however, sCD163 remained chronically elevated. The same study also found plasma soluble CD14 levels elevated in individuals with chronic HIV infection, before and after cART initiation, compared with HIV-seronegative controls. Both molecules, sCD14 and sCD16, have been found to play a role in atherogenesis in PLWH (62).

Continuous, cumulative exposure to noxious viral particles and inflammatory signals over time, which happens even while in cART, may damage the endothelium, increasing its permeability and promoting leucocyte invasion into the vessel wall. A chronic inflammatory state may then set the stage for arterial wall remodeling. Circulating HIV protein Tat, for example, has been found to cause coronary endothelial dysfunction and non-compliance, oxidative stress and disruption of brain microvascular endothelial function, in animal models and in humans (63, 64). In a macaque model, Simian Immunodeficiency Virus protein Nef was associated with a range of pathological vascular phenotypes, from medial hypertrophy to thrombosis (65, 66). In a porcine model, HIV protein Nef was shown to decrease endothelium-dependent vasorelaxation in pulmonary arteries (66). Both Nef and Tat have been associated with increases in endothelial apoptosis, angiogenesis, inflammatory cytokines and cell adhesion molecules (67). During initial infection, before viremia can be suppressed by cART, increased circulation of viral proteins and active inflammation may lead to a more rapid and dramatic remodeling of vascular architecture (68).

**Figure 1:**
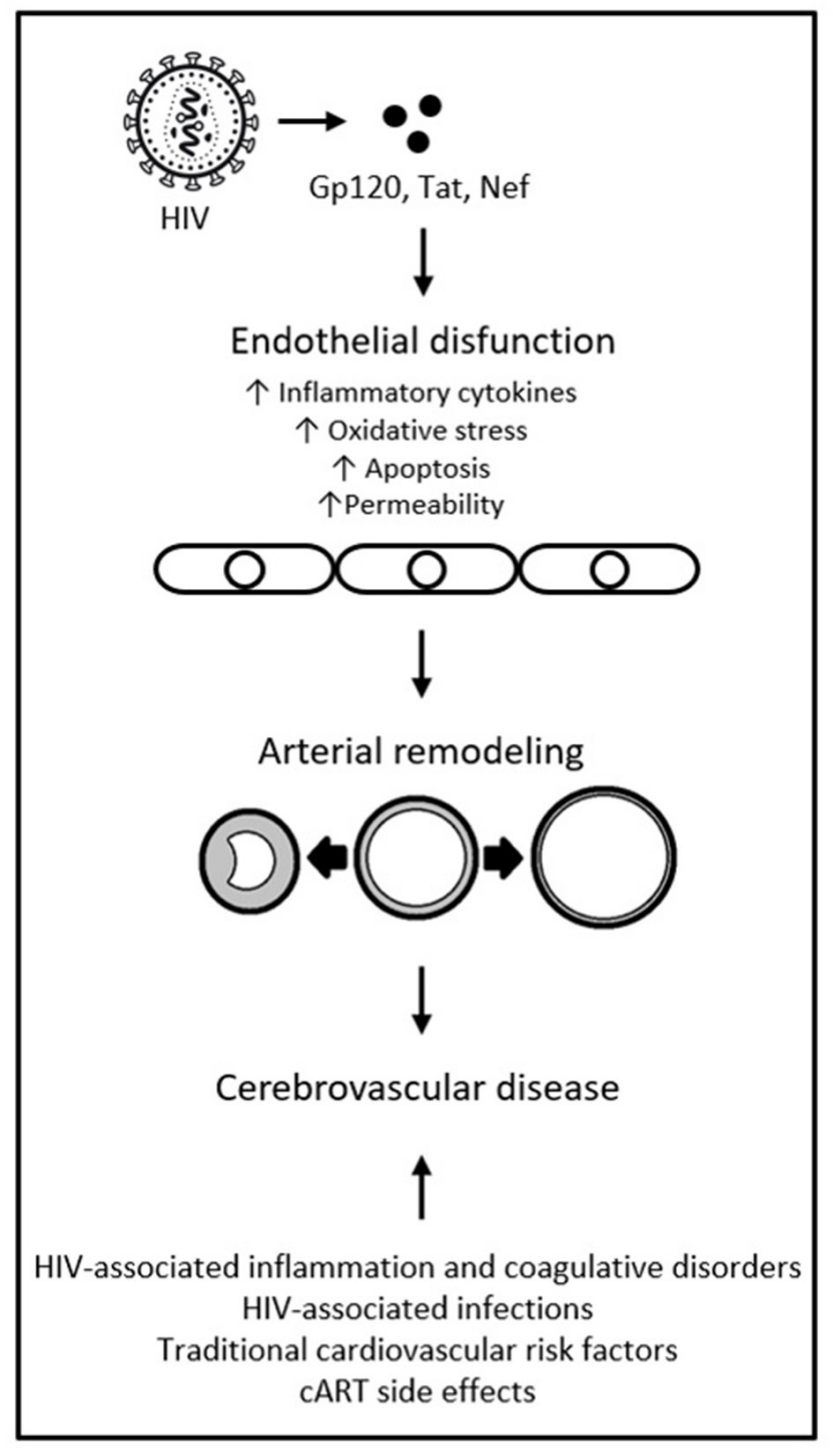
HIV-coded proteins as a cause of endothelial dysfunction and arterial remodeling. Adapted from “HIV proteins and endothelial dysfunction: implications in cardiovascular disease,” by A. R. Anand, G. Rachel and D. Parthasarathy, 2018, *Frontiers in cardiovascular medicine*, 5, p. 185 (https://www.frontiersin.org/articles/10.3389/fcvm.2018.00185/full). CC BY.

Viral protein Gp120, in both soluble and surface-bound forms, has also been shown able to alter function of bystander cells that are not directly infected with HIV, including endothelial cells. It has been associated with endothelial cell apoptosis, adhesion molecule expression, production of inflammatory cytokines, increased expression of matrix metalloproteinases, and increased permeability (69–73). A study found Gp120 to promote endothelial cell senescence in humans; a phenotype that promotes inflammation, vasoconstriction, and thrombus formation (74, 75). The chronic effects of HIV proteins on the endothelium may be intertwined with (and confounded by) the effects of concomitant cART. cART-induced endothelial damage has been shown to play a role in the mechanism of endothelial dysfunction and cerebrovascular risk. However, the deleterious effects of cART are mostly thought to occur through metabolic abnormalities, not direct endothelial damage (2, 55, 76). While some specific ART classes (e.g., protease inhibitors) and individual drugs (e.g., abacavir) have been associated with increased cerebrovascular risk, current cART regimes are largely believed to reduce the risk overall, not increase it (42, 76). There is also variability in the ability of different antiretrovirals to reach therapeutic concentrations in cerebrospinal fluid (CSF): under suboptimal drug pressure, continuous replication of HIV in the central nervous system (CNS) is possible (77). This is evidenced by CSF viral escape, in which HIV RNA can be detected in CSF when it is undetectable in plasma. CSF viral escape occurs in 4 to 20% of PLWH, has been associated with cART regimes of protease inhibitors and nucleoside reverse transcriptase inhibitors, and with CNS inflammation (78, 79). This exposes the cerebrovascular endothelium to higher concentrations of viral particles and proteins, despite systemic viral suppression. It is possible that HIV-coded proteins interact with traditional risk factors, chronic inflammation, and cART, to ultimately cause endothelial dysfunction. This, in turn, may lead to pathological phenotypes of arterial remodeling and ultimately cerebrovascular disease, as represented in Figure 1 (67). Such interactions are sure to be complex, however, and much remains to be discovered about them.

## HIV-Associated Inward Arterial Remodeling

Compared to HIV-seronegative controls, PLWH in long-term cART continue to show increased rates of atherosclerosis, an extreme phenotype of inward remodeling, with associated increase in risk of acute ischemic events (80, 81). The current body of knowledge of HIV-associated atherogenesis has been gained mostly through clinical studies, with very few experimental studies to help explain the mechanisms for this association, although it is well-accepted that HIV infection promotes accelerated atherosclerosis in extra and intracranial arteries (57, 82). A 2015 US-based brain bank study described a direct association between HIV status and inward remodeling of intracranial arteries (57). They observed that, compared with HIV-seronegative controls, PLWH had a predisposition for inward remodeling of brain large arteries, with thicker media, thicker arterial walls, and smaller lumen-to-wall ratio. These associations were found after adjusting for vascular risk factors, with no statistical difference in stenosis or calcification. With a sample of 142 HIV-positive and 142 HIV-seronegative brain donors, half of all brain infarcts among the PLWH in that study were attributed to one of two arterial remodeling extremes: atherosclerosis or dolichoectasia. Atherosclerosis accounted for a quarter of brain infarcts in the study's PLWH sample. Intracranial atherosclerosis was significantly associated with a lower CD4 nadir and a higher antemortem CD4 count. This, the authors noted, was a novel finding, and one which agreed with HIV-associated changes that had been reported in extracranial arteries by other studies (83, 84). It suggested a role for the immune system in the development of atherosclerosis, one in which a bigger difference between CD4 count before and after successful cART results in greater vascular inflammation. Successfully treated HIV infection with immune reconstitution could therefore be associated with higher rates of inward remodeling of intracranial arteries, compared to immunocompromised PLWH (who would present a different remodeling phenotype) and to HIV-seronegative controls. The exact mechanisms linking inward remodeling and ultimately atherosclerosis with HIV, however, remain unexplained.

In a another brain bank study, from 2018 (85), it was observed that intimal lymphocytic inflammation was involved in brain arterial remodeling, possibly contributing to the cerebrovascular pathological findings in that PLWH sample. The authors analyzed large brain arteries from 84 PLWH and 78 HIV-seronegative controls. In brain samples of PLWH with antemortem CD4 count over 200, and of HIV-seronegative controls, a higher number of CD3 T cells infiltrating the intima was associated with histological markers of hypertrophic inward remodeling. In samples from PLWH with CD4 counts less than 200, however, the presence of CD3 T cells in the intima was associated with hypertrophic outward remodeling instead. The researchers hypothesized that “a sufficient” CD3 T cell count may be needed to generate an inflammatory response that leads to inward remodeling in HIV, with subsequent luminal and blood flow reductions. The authors also reported that adventitial CD3 T cells were decreased among PLWH compared to HIV-seronegative controls. The decrease was more pronounced in samples belonging to immunosuppressed PLWH. The CD3 T cell numbers in the intima, however, did not differ by HIV status, as they did in the adventitia, and adventitial CD3 T cells were not associated with atherosclerosis. The authors of that study could not elucidate the exact role of CD3 T cells in brain arterial remodeling, as that marker comprises a variety of cell subpopulations with different functions. Their results, however, pre-suppose that inflammatory cells in the brain of PLWH may affect distinct arterial layers differently, and immune cell quality and quantity in each layer may be associated with immune status and disease history. It is possible that CD3 T cells are involved in brain inflammatory changes such as HIV-associated vasculitis, arterial dilatation and inflammation limited to the CSF (86, 87). The use of a semi-quantitative measurement of CD3 T cells, and the fact that all HIV-positive brain samples proceeded from the same site, were limitations to this study. Its results nonetheless point to the existence of differential markers of inflammation in, and differential remodeling of, brain arteries in immunocompromised PLWH when compared to immunocompetent PLWH and to HIV-seronegative individuals.

Biomarker studies indicate that there may also be a role for monocyte-macrophage activation in HIV-associated atherosclerosis. A study using a transgenic mouse model (88) showed that, in mice with an ApoE^−/−^ phenotype who also expressed HIV-1, HIV expression was enough to accelerate atherosclerosis, with increased caspase-1 pathway activation in inflammatory monocytes. The same study also analyzed *in vivo* samples from PLWH and postmortem samples from an HIV-positive human tissue bank. The authors documented that *in vivo* plasma IL-18 was higher in PLWH compared with HIV-seronegative controls. Higher IL-18 levels were associated with markers of monocyte-macrophage activation and non-calcified, inflammatory coronary plaques. In the postmortem tissue sample of PLWH, aortic plaques were associated with caspase-1-positive cells and CD 163-positive macrophages. This study demonstrated that exposure to HIV may independently accelerate atherogenesis in humans. It also highlighted the possible role of the caspase-1 pathway and of monocyte-macrophage activation in HIV-associated atherogenesis.

Cerebrovascular disease mechanisms in PLWH may also vary according to CD4 count. In a retrospective study of PLWH (89) it was seen that, among 115 stroke cases, most (22%) were due to large artery atherosclerosis (17%, due to small artery disease; 16%, infectious; 8%, cardioembolic; 21%, cryptogenic; and 16%, other causes). They found that large artery atherosclerosis was significantly associated with longer HIV infection and CD4 nadir less than 200. In the same sample, stroke due to large artery atherosclerosis was associated with higher CD4 count in the year prior to stroke, independent of CD4 nadir. They concluded that large artery atherosclerosis was the most frequent stroke mechanism in PLWH whose nadir CD4 count was less than 200 (which suggests cART start later in infection history) and whose CD4 count near the time of the stroke was higher (which suggests successful cART). These *in vivo* results support those of the brain bank study described above (85), where hypertrophic inward remodeling, of which atherosclerosis constitutes an extreme, was most frequently seen in brain arteries of PLWH with higher antemortem CD4 count.

**Figure 2:**
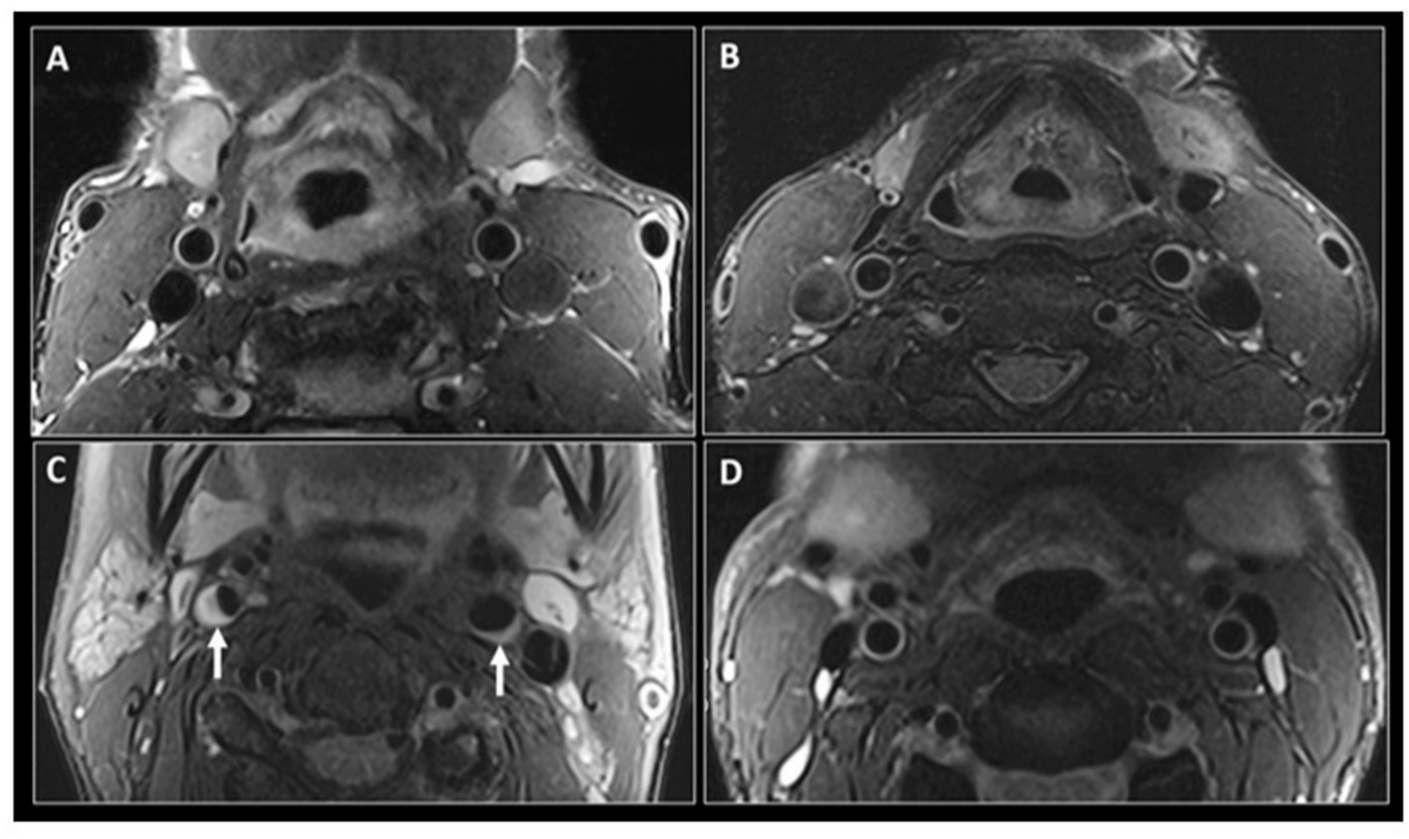
Black-blood MR imaging of the carotid arteries. Adapted from “Subclinical atherosclerosis imaging in people living with HIV,” by I. C. Schoepf, R. R. Buechel, H. Kovari, D. A. Hammoud and P. E. Tarr, 2019, *Journal of clinical medicine*, 8(8), p. 1125 (https://www.mdpi.com/2077-0383/8/8/1125). CC BY. Fat saturated T2-weighted black-blood MR images at the level of the common carotid arteries in a 56-year-old HIV-positive man **(A)** and a 47-year-old HIV-negative man **(B)**. Similar imaging technique at the level of the internal carotid arteries in a 56-year-old HIV-positive woman **(C)** shows narrowing of the vascular lumen bilaterally by a plaque (small arrows), more significant on the right side. **(D)** shows similar imaging at the level of internal carotid arteries in a 47-year-old HIV-negative man with no evidence of atherosclerosis.

Detecting HIV-associated brain arterial remodeling in PLWH, *in vivo*, however, remains a challenge. Imaging has a limited diagnostic or prognostic role in HIV-associated arterial remodeling. But this role may be expanded in the future, with the advent of more advanced or specific imaging procedures. Black-blood MRI (BBMRI), for example, is an advanced technique that allows better visualization of the vascular wall thickness by nulling the signal from the vascular lumen. In current practice, it is mostly used to assess visual markers of cardiovascular and cerebrovascular risk from eccentric lipid-rich plaques (90). But in PLWH it could be used to measure the vessel wall thickness (91, 92), as seen in the example presented in Figure 2, abstracted from the 2019 study “Subclinical Atherosclerosis Imaging in People Living with HIV” (93). In an imaging study with subjects with low traditional cardiovascular risk, HIV-status was significantly associated with increased vascular thickening, after adjusting for age (92). In another study, PLWH on cART also showed increased carotid artery wall thickness on MR imaging compared to HIV-seronegative controls with similar cardiovascular risk (94). In sum, the full potential of MRI techniques to measure arterial remodeling in PLWH in a way that could be clinically relevant remains undefined, but promising.

## HIV-Associated Outward Arterial Remodeling

While inward remodeling leads to vessel stenosis and atherosclerosis, outward remodeling is usually accompanied by media thinning and vessel dilation (4, 95). In the same US-based 2015 brain pathology study described above (57), the researchers found that outward remodeling was the “defining arterial phenotype” among PLWH that experienced prolonged immunosuppression prior to death. They correlated dolichoectasia (an extreme outward remodeling phenotype) with media thinning and higher viral load at the time death. Furthermore, higher lumen-to-wall ratio was the only arterial remodeling variable associated with cryptogenic brain infarcts in their PLWH sample. This somewhat confounded their other finding that inward remodeling was linked to HIV, albeit in HIV-positive cases with higher antemortem CD4 counts. The authors posited that immune reconstitution (with increased numbers of CD3 T cells) would generate a different and more robust inflammatory response, leading to a different remodeling phenotype, than what is seen in the immunosuppressed. This hypothesis, however, is yet to been tested.

In the other US-based brain bank study, from 2018 (85), the authors found that intimal lymphocytic inflammation with hypertrophic outward remodeling was associated with adventitial macrophages and increased elastolysis activity. They described similar findings in a separate study of the same cohort (96). This association has also been documented in extracranial arteries, such as in aortic and coronary aneurysmal dilatations (97–99). The authors of the 2018 brain bank study (85) proposed that an interaction between immunosuppression and intimal CD3 T cells may potentiate arterial dilatation, rather than constriction. Adventitial CD3 T cell expression was not associated with intracranial large artery atherosclerosis in their sample. Consequently, they proposed that inflammatory cells “may affect the intima differently than the adventitia of brain arteries.” The exact role of adventitial CD3 T cells in arterial inflammation or remodeling is yet to be described, but it is certainly possible that these cells are involved in other inflammation-induced vascular changes. Indeed, separate studies have associated the presence of CD3 T cells with HIV-associated vasculopathy and arterial dilatation (86, 87). These inflammatory cells could contribute to adventitial inflammation in PLWH while having a different role in the intimal inflammatory process that leads to stenosis or atherosclerosis.

Evidence is accumulating in support of an independent role for HIV in the pathogenesis of vasculopathy. In the current literature, HIV-associated vasculopathy conventionally refers to abnormalities of blood vessels that are a direct or indirect consequence of HIV infection, with no alternative etiological explanation (42). This diagnosis is usually reserved for PLWH who present with clinical or radiological features of vasculopathy and in whom other causes have been ruled out (2). In past clinical case series, 13 to 28% of ischemic strokes in PLWH had been attributed to HIV-associated arterial vasculopathy. Most of these studies, however, did not rule out vasculopathy attributable to opportunistic infection (3, 100, 101). Evidence of vasculopathy has also been found in postmortem brain samples of PLWH, even in subjects with long-term successful viral suppression before death (102, 103). Animal models have directly linked HIV infection to vasculopathy, independently from other causes. For example, a study of the murine AIDS model in mice suggested that retroviral infection can cause endothelial dysregulation and vasculopathy. Similar experimental findings have been corroborated in humans (104–106). Outward remodeling with thinning of the arterial media layer has been reported as a possible pre-clinical stage for HIV vasculopathy (38). HIV infection could be initiating vascular injury in the brain, or perhaps contribute to further injury to vasculature already damaged by atherosclerosis, pre-disposing PLWH to stroke. The exact mechanism of arterial wall damage in the context HIV remains poorly understood, especially among subjects on long-term cART. But pathological data suggests that, among people who died with HIV, antemortem low CD4 counts and low CD4 nadir were associated with intracranial arterial outward remodeling involving wall thinning and arterial dilatation (57).

*In vivo* imaging may also be an important tool in assessing outward remodeling phenotypes in brain arteries associated with HIV infection. A 2019 MRI study (68) using T2-weighted imagining sequences, found that the vascular caliber of the anterior cerebral artery, A1 segment, was higher in PLWH compared to HIV-seronegative controls, matched for sex and race. Meaningfully, higher CD4 T cell count and longer duration of infection were associated with decrease in A1 caliber of PLWH. The findings of this study were in agreement with postmortem observations in brains of PLWH (5, 38, 40). It is therefore possible that the observed MRI changes in lumen caliber would reflect HIV-associated vessel wall thinning and/or loss of compliance with protracted infection. A brain bank study recognized an association between HIV infection and thinning of medial arterial layers, which may be a pre-clinical stage in HIV-related vasculopathy (38). Other studies have established adventitial inflammation in the context of HIV infection is associated with a thinner media, and outward remodeling of the arterial wall that would ultimately lead to dolichoectasia (40, 107). Chronic HIV-induced damage to the inner endothelial layer could affect vascular compliance, which could at least partially explain the arterial luminal changes observed in brain MRI studies of PLWH (59, 68, 108).

## Cerebrovascular Remodeling and HIV-Associated Neurocognitive Disorders in the cART ERA

HIV-associated neurocognitive disorders (HAND) refer to a wide range of neuropsychological impairments in the context of HIV infection. While the cellular and physiological mechanisms that lead to HAND remain poorly understood, they likely involve chronic neuroinflammation and have the potential to alter cerebrovascular architecture (109–111). HIV encephalitis and widespread neuronal loss, previously thought to have pivotal roles in the development of HAND, are no longer typically seen in PLWH on long-term, stable cART who present with neurocognitive impairments (112). While a recent study of histopathological phenotypes associated with HAND showed that pre-synaptic degeneration may precede somatodendritic degeneration and lead to neurocognitive impairment (113), the study cohort was composed of individuals with more advanced illness and high frequency of HIV encephalitis, which may not be reflective of the growing PLWH population who remain in long-term cART (114). The introduction of cART, in fact, reduced the overall frequency of HIV encephalitis from 54% to less than 15% (115, 116). However, markers of HIV-induced inflammation in the CNS are still present after viral replication has been suppressed by cART (117, 118). The post-cART pathology of HAND seems to have shifted to subtler, chronic neurodegeneration, affecting more cortical regions (119).

The effects of aging, chronic HIV infection and chronic cART may interact and cause neurodegeneration. These mechanisms include neuroinflammation, oxidative stress, DNA damage, cell senescence and defective proteostasis (proteasome, proteolysis and autophagy disfunctions) (120). These common alterations may be synergistic and lead to abnormal accumulation of proteins typically involved in neuronal damage and dementia (Amyloid β, Tau, α-synuclein). Dementia cases associated with HIV in the post-cART era usually also show diffuse astrogliosis, microglial nodules, white matter alterations and vascular changes with peri-vascular lymphocytic infiltration (121). Due to the several common and overlapping molecular markers involved, trying to differentiate HAND from the mechanisms of normal aging, Alzheimer's disease, vascular and other forms of dementia continues to be a source of controversy and debate (122). Vascular disease of the brain, however, is almost universally thought to play a role in post-cART HAND (6, 114, 123, 124).

Neuroinflammation associated with HIV infection has the potential to compromise normal cerebrovascular function. While HIV-induced intracranial large artery atherosclerosis would restrict blood flow, the infection may also cause cerebral small vessel disease (CSVD), further affecting cerebral perfusion (15, 125–130). While CSVD in the general population is largely associated with hypertension, diabetes, and aging (131), PLWH on cART seem to be at even higher risk, unexplained by those exposures alone. A French study, for instance, revealed that PLWH with well-controlled infection had twice the prevalence of silent CSVD as uninfected controls (129). The authors put forward HIV infection as an independent risk factor for CSVD. An American study, on the other hand, suggested that HIV infection and CSVD are independent, additive processes that together cause brain atrophy and cognitive impairment (132). The direct effects of HIV on cerebral vessels are also difficult to separate from the potential toxic effects of long-term cART. A US-based cross-sectional study, for instance, showed an association between CSVD and cART regimes that include protease inhibitors (133), after adjusting for diabetes. Mild CSVD itself, the same study showed, was associated with HAND.

Irrespective of the specific mechanisms, post-cART HIV infection increasingly seems associated with cerebrovascular dysregulation and, ultimately, vascular remodeling leading to neurocognitive dysfunction, especially in aging PLWH (116, 134). Small and large intracranial vessel remodeling, particularly atherosclerosis for those on long-term cART, could be contributing factors to cognitive impairment in older PLWH (124). In a 2014 US-based cohort study, PLWH who were over 50 years of age were twice as likely as younger PLWH to have HAND, even after adjusting for dementia risk factors (135, 136). A similar risk disparity has been observed in South African aging cohorts (137, 138). HAND risk increases seen with age, however, remain confounded by the increasing prevalence of cerebrovascular risk factors in aging PLWH (139, 140). A 2010 US cohort study of 1,555 adult PLWH, for instance, found that older age, elevated blood pressure, BMI, high cholesterol, and a prior diagnosis of AIDS were all associated with worse neuropsychological performance (141).

A 2016 American cohort study of 197 PLWH showed that only 10% had a measurable improvement of HAND after cART introduction, with 77% remaining more or less neurocognitively stable and 13% deteriorating to more severe HAND while on cART (142). The START trial, on the other hand, failed to confirm major improvement of HAND brought on by early cART (14, 140). HAND, for the most part, does not seem to progress in most PLWH on stable cART after immune reconstitution. Still, for patients diagnosed with HAND who initiate cART, HAND rarely resolves completely. Some alterations in brain function induced by HIV infection may therefore be structural, longer lasting and/or unpreventable even by stable cART. In a US-based 2019 brain bank study that included 94 PLWH, researchers performed antemortem measures of motor functioning, processing speed, working memory, verbal fluency, and executive functioning (143). They reported an association between brain arterial wall thickening and poorer global cognitive score, processing speed and verbal fluency. Associations were independent of traditional vascular risk factors, CD4 count, viral load, or cART use. Intracranial arterial wall thickening was also associated with both incident and, more strongly, with worsening HAND at the time of death. The effects that classic vascular risk factors have on cognitive performance appear greater in studies of cohorts with higher CD4 counts (143, 144). The association between classical vascular risk factors and cognitive performance may therefore be outweighed by the effects of persistent immunosuppression.

Even accounting for limitations in sample size and biases inherent to autopsy series, the 2019 brain bank study mentioned above (143) signals a potential role for arterial remodeling in HIV-associated neurocognitive decline. This role was studied *in vivo* in a 2018 US-based cross-sectional analysis of 72 PLWH and 36 HIV-seronegative controls, all over 50 years of age (111). The authors found an association between markers of vascular remodeling (specifically, lower Tie-2, and higher VEGF) and worse neurocognitive function only in PLWH, suggesting that HIV infection moderates this association. Variables other than HIV itself linking arterial remodeling with HAND, however, cannot been ruled out. Brain arterial wall thickening is, after all, naturally associated with aging, Alzheimer's disease and other forms of dementia (145). Moreover, while atherosclerosis is a frequent phenotype of arterial inward remodeling, it is not the only one (146, 147). Compensatory intimal thickening with no atheroma may result from the normal aging process, resulting in wall thickening and arterial stiffness (145, 148). Diabetes, also associated with HAND, could likewise confound the association between arterial remodeling and cognitive scores (149, 150). In the same 2019 brain bank study from above (143), possible mechanisms linking viral suppression, lumen preservation, and cognition remained undetermined. That viral suppression and cART use were both associated with larger luminal diameters and better cognition was, nonetheless, highly suggestive (143, 151). It is also possible that HIV-associated inflammation may act as an effect modifier in the association between intracranial arterial wall thickness and cognition, as opposed to having an individual causal effect. Further studies measuring systemic inflammation and cerebrovascular pathology would be necessary to test if this is the case. Still, there is accumulating evidence of a possible role for HIV-induced vascular remodeling in the development and progression of HAND post-cART initiation, a role that should be further explored.

While more severe forms of HAND have become rare post-cART (43), there is evidence for widespread vascular cognitive impairment (VCI) in aging PLWH, albeit in milder and subclinical forms (129, 143, 152, 153). VCI refers to all types of cognitive disorder associated with cerebrovascular disease, regardless of specific mechanisms (154). It comprises cognitive deficits ranging from mild cognitive impairment to dementia. While the neuropsychological and neuroimaging phenotypes of VCI and HAND are largely overlapping and may even represent aspects of the same neuropathological entity for PLWH, the literature mostly describes them independently (43). In PLWH on long-term cART, mild forms of both HAND and VCI are associated with persistent subclinical or clinical cerebrovascular disease, with HIV acting as a vascular risk factor (34, 155, 156). Further studies have posited that HIV-induced chronic immune activation, immune senescence, viral reservoir activity, microbial translocation, and reactivation of its commonly associated pathogens (such as CMV and herpes simplex) are also involved in mild HAND and VCI (112, 114, 157, 158).

Although the exact causes remain unknown (and are sure to be multifactorial and complex), both VCI and HAND are thought to be, at least partially, generated and/or worsened by HIV-induced cerebrovascular disease (110, 159). HIV-induced intracranial arterial remodeling may therefore play a pathogenic role in both entities. Some researchers have put forward a new hypothesis proposing that the neurovasculature may actually be a primary target for chronic HIV injury (112, 160). Endothelial cell surfaces, they propose (and we have discussed), are chronically perturbed in PLWH who are successfully treated and virally suppressed. This, they posit, leads to chronic alteration of the neurovascular unit, altering the brain's arteries, microvasculature and, subsequently, blood perfusion. Results from the National NeuroAIDS Tissue Consortium brain gene array study may back up this theory (160). The authors of that study found that HAND (without HIV encephalopathy) is characterized by abnormal regulation of gene transcription in brain endothelial cells (161). This chronic alteration of neurovascular biology may be a prevalent process in virally suppressed PLWH. Neurovascular unit damage-associated forms of VCI and HAND could indeed be the more prevalent forms in the post-cART. This would help explain the milder clinical profile of cognitive impairment in cohorts of PLWH on long-term, successful cART, in whom acute inflammatory infiltrates of the brain are rarely seen, while low-grade chronic immune activation is much more prevalent (6, 123, 162–165). In the patients who remain immunocompromised, however, acute inflammation would continue to be the more prevalent mechanism of injury to brain vasculature (112, 160).

## Considerations Regarding SARS-COV-2 and HIV Coinfection

The coronavirus disease 2019 (COVID-19) pandemic is caused by the severe acute respiratory syndrome coronavirus 2 (SARS-CoV-2). This virus binds to the angiotensin-converting enzyme 2 (ACE2) to infect cells (166, 167). This enzyme is expressed in the lungs, small intestine and brain (168). The expression of ACE2 in cortical neurons and glia makes them susceptible to SARS-CoV-2, which may explain the high incidence of anosmia and other neurological deficits seen in COVID-19 (169). In the brain, however, ACE2 is also expressed in endothelial and smooth muscle cells. ACE2 normally counteracts the effects of ACE1 and angiotensin II. Overexpression of ACE2 in neuronal cells and endothelial progenitor cells, in fact, has a protective effect from ischemic stroke (170, 171). As SARS-CoV-2 invades cells it depletes ACE2 through receptor endocytosis, leaving ACE1 unopposed. Resulting angiotensin II excess impairs endothelial function, leading to a proinflammatory state with organ-damaging effects seen in the lungs, heart and brain (172). SARS-CoV-2 proliferation in vascular endothelial cells also leads to endothelialitis (173). SARS-CoV-2 antigens can activate the complement system, macrophages, and neutrophils, further damaging endothelial cells. This injury may compound the loss ACE2's vasoprotective effects (174). This is particularly concerning given that even limited injury to the endothelium of cerebral vessels can initiate *in situ* thrombosis and lead to strokes (175, 176).

Recent retrospective studies of PLWH hospitalized due to COVID-19 conducted in the US, however, found no significant differences in clinical outcomes of PLWH compared to HIV-seronegative controls (177, 178). Cohort studies conducted in Spain similarly reported a lack of significant differences in COVID-19 hospitalization outcomes for the two groups (179, 180). One of those studies further noted that PLWH receiving cART regimes that included tenofovir disoproxil fumarate with emtricitabine had indeed lower risk of COVID-19 infections and hospitalizations, compared to PLWH receiving other cART regimes (180). But these results remain in need of confirmation in other populations. While possible interactions of SARS-CoV-2 and HIV are actively being researched, the possible long-term effects remain unknown, especially as it relates to PLWH on long-term cART. It is possible that SARS-CoV-2 may compound endothelial damage brought on by chronic HIV infection in the brain vasculature, with certain cART regimes offering more protection from these effects. Nevertheless, the research on SARS-CoV-2 infection in PLWH is still too nascent to offer any conclusions.

## Discussion

The vast majority of PLWH reside in low-to-middle-income countries, where overall stroke incidence has more than doubled in the last 40 years (181, 182). It is thus expected that global stroke incidence in PLWH will continue to increase (183). In high-income countries, on the other hand, the absolute numbers of stroke in PLWH on cART are comparatively low (42). Nonetheless, the relative rise in HIV-associated ischemic stroke post-cART introduction is still a public health concern. Therefore, developing the capacity to prevent cerebrovascular morbidity and mortality in an aging PLWH population constitutes an increasingly urgent public health priority, for both low-to-middle- and high-income countries.

The literature reveals that PLWH on cART still suffer higher rates of cerebrovascular disease than the general population (10–13). Stroke in PLWH occurs pre-maturely and is less associated with traditional risks factors compared to HIV-seronegative controls (11, 12, 32, 33). Ischemic stroke is the type most often associated with HIV in the post-cART era, with hemorrhagic stroke remaining the more frequent form in immunocompromised PLWH (3, 22, 23, 184). But significant gaps in the literature remain regarding the specific pathophysiology of cerebrovascular disease in PLWH. These gaps may preclude health providers and researchers from more accurately assessing and preventing cerebrovascular risk in PLWH, compared to HIV-seronegative populations. Still, considering the available data, emphasizing cardiovascular risk reduction interventions to optimize cardiovascular health is essential for maintaining brain health in an aging PLWH population. Such interventions may mitigate the effects of HIV-associated pathological cerebrovascular remodeling, when combined with appropriate and sustained cART (132).

Increasing evidence shows that vascular endothelium is affected by circulating HIV products in the context of long-term cART, even with low or undetectable viremia and no discernible direct interaction between endothelial cells and the virus (51–53). HIV-induced endothelial dysfunction is a likely precursor to arterial remodeling. The endothelium may initiate and propagate atherogenesis while also inducing thrombus formation, pre-disposing PLWH to ischemic stroke (2, 57). In order to minimize the effects of HIV in brain vascular endothelium, cART regimes that are more likely to reach and maintain therapeutic concentrations in the CNS should be favored. Still, the complex interactions between HIV-infection, circulating HIV particles, cART, and traditional cerebrovascular risk factors leading to arterial remodeling remain poorly understood. Additionally, endothelial damage induced by of SARS-CoV-2 and HIV coinfection, in the context of the COVID-19 pandemic, is a possibility. This and other possible long-term effects of the COVID-19 pandemic on PLWH on cART, however, remain to be seen.

HIV infection is associated with inward remodeling in general, and atherosclerosis in particular, of intracranial arteries (57, 82). Because lower CD4 nadir is associated with intracranial large artery atherosclerosis, even after prolonged immune reconstitution brought on by cART (89), proper population screening leading to early HIV diagnosis is essential. Early diagnosis would allow for the start and maintenance of cART before an accentuated drop in CD4 occurs, which could potentially help prevent brain atherosclerosis associated with a lower CD4 nadir.

Arterial remodeling may also play a role in HAND, especially in the milder forms which patients on stable cART more often express (116, 134). Both small and large vessel atherosclerosis have been linked to cognitive impairment in older PLWH (124). Long-term cART and viral suppression, on the other hand, were associated with larger intracranial arterial vessel diameters and better cognition (143, 151). The current literature shows that treated HIV infection is associated with premature aging, which affects the brain (128). However, the extent of the overlap between HIV-induced changes in the brain of PLWH, non-HIV types of dementia, and normal aging, remains a matter of debate. While the exact mechanisms through which PLWH on cART develop VCI and HAND are unknown, they are at least partially originated or worsened by intracranial cerebrovascular remodeling (110, 159). Interventions aimed at preventing pathological brain vascular remodeling may, therefore, have some positive effects on the overlapping forms of HIV-associated cognitive decline post-cART. Such interventions and their effects are also targets for future research.

Neuroimaging may have a role to play in the future of cerebrovascular risk assessment and prevention in HIV infection. MRI techniques have been able to detect the arterial wall thickening and atherosclerosis associated with treated HIV infection (91, 92). MRI also showed some promise in measuring HIV-associated vasculopathy *in vivo*. A recent imaging study, for instance, showed that anterior cerebral artery caliber was higher in PLWH compared to controls, but higher CD4 T cell count and longer-treated HIV infection were associated with decreases in that same caliber (68). MR imaging may therefore be used in the future to assist in elucidating the natural history of arterial remodeling in successfully treated HIV infection, but the current literature on this subject remains limited and inconclusive. The clinical and screening applications of imaging these for the benefit of PLWH cerebrovascular health remains to be tested.

No pharmacological interventions were found in the literature that would significantly reverse HIV-associated pathological brain arterial remodeling. For the general population, statin therapy has shown some effect on improving pathological remodeling phenotypes and atheroma composition, leading to modest improvement of microvascular function in coronary artery disease (185). Statins have also shown some protective effects against stroke and other embolic events in patients with aortic atherosclerotic plaques (186). Therapeutics that would reverse pathological arterial remodeling in the brain of PLWH, however, have not been studied. Therefore, it is recommendable that effective HIV long-term care continues to be accompanied by standard cardiovascular risk prevention, which has the potential to impede the progression of pathological vessel remodeling. More research leading to an improved understanding of brain arterial remodeling phenotypes associated with HIV may reveal further therapeutic targets. These targets would present opportunities to reduce the burden of cerebrovascular disease and cognitive impairment in the aging population of PLWH on cART.

## Author Contributions

AS-A reviewed the literature and co-wrote the manuscript. JG co-wrote the manuscript.

## Conflict of Interest

The authors declare that the research was conducted in the absence of any commercial or financial relationships that could be construed as a potential conflict of interest.

## References

[1] Deeks SG. Immune dysfunction, inflammation, and accelerated aging in patients on antiretroviral therapy. Top HIV Med. (2009) 17:118–23. 19890183

[2] Benjamin LA Bryer A Emsley HC Khoo S Solomon T Connor MD. HIV infection and stroke: current perspectives and future directions. Lancet Neurol. (2012) 11:878–90. 10.1016/S1474-4422(12)70205-3 22995692 PMC3460367

[3] Tipping B de Villiers L Wainwright H Candy S Bryer A. Stroke in patients with human immunodeficiency virus infection. J Neurol Neurosurg Psychiatry. (2007) 78:1320–4. 10.1136/jnnp.2007.116103 17470469 PMC2095583

[4] Qiao Y Anwar Z Intrapiromkul J Liu L Zeiler SR Leigh R. Patterns and implications of intracranial arterial remodeling in stroke patients. Stroke. (2016) 47:434–40. 10.1161/STROKEAHA.115.009955 26742795 PMC4729583

[5] Gutierrez J Elkind MS Petito C Chung DY Dwork AJ Marshall RS. The contribution of HIV infection to intracranial arterial remodeling: a pilot study. Neuropathology. (2013) 33:256–63. 10.1111/j.1440-1789.2012.01358.x 23067346 PMC4388028

[6] Brew BJ. Has HIV-associated neurocognitive disorders now transformed into vascular cognitive impairment? AIDS. (2016) 30:2379–80. 10.1097/QAD.0000000000001225 27603161

[7] Sabin CA. Do people with HIV infection have a normal life expectancy in the era of combination antiretroviral therapy? BMC Med. (2013) 11:251. 10.1186/1741-7015-11-251 24283830 PMC4220799

[8] Teeraananchai S Kerr SJ Amin J Ruxrungtham K Law MG. Life expectancy of HIV-positive people after starting combination antiretroviral therapy: a meta-analysis. HIV Med. (2017) 18:256–66. 10.1111/hiv.12421 27578404

[9] Sabin C. Review of life expectancy in people with HIV in settings with optimal ART access: what we know and what we don't. J Int AIDS Soc. (2012) 15:18076. 10.7448/IAS.15.6.18076

[10] Smith CJ Ryom L Weber R Morlat P Pradier C Reiss P. Trends in underlying causes of death in people with HIV from 1999 to 2011 (D:A:D): a multicohort collaboration. Lancet. (2014) 384:241–8. 10.1016/S0140-6736(14)60604-8 25042234

[11] Quiros-Roldan E Raffetti E Foca E Brianese N Ferraresi A Paraninfo G. Incidence of cardiovascular events in HIV-positive patients compared to general population over the last decade: a population-based study from 2000 to 2012. AIDS Care. (2016) 28:1551–8. 10.1080/09540121.2016.1198750 27321070

[12] ChowFC. HIV infection, vascular disease, and stroke. Semin Neurol. (2014) 34:35–46. 10.1055/s-0034-137234124715487

[13] Ovbiagele B Nath A. Increasing incidence of ischemic stroke in patients with HIV infection. Neurology. (2011) 76:444–50. 10.1212/WNL.0b013e31820a0cfc 21248273 PMC3034413

[14] INSIGHT START Study Group Lundgren JD Babiker AG Gordin F Emery S Grund B. Initiation of antiretroviral therapy in early asymptomatic HIV infection. N Engl J Med. (2015) 373:795–807. 10.1056/NEJMoa1506816 26192873 PMC4569751

[15] Kearns A Gordon J Burdo TH Qin X. HIV-1–associated atherosclerosis: unraveling the missing link. J Am Colle Cardiol. (2017) 69:3084–98. 10.1016/j.jacc.2017.05.012 28641798 PMC5512584

[16] Kieburtz KD Eskin TA Ketonen L Tuite MJ. Opportunistic cerebral vasculopathy and stroke in patients with the acquired immunodeficiency syndrome. Arch Neurol. (1993) 50:430–2. 10.1001/archneur.1993.00540040082019 8460966

[17] Berger JR Harris JO Gregorios J Norenberg M. Cerebrovascular disease in AIDS: a case-control study. AIDS. (1990) 4:239–44. 10.1097/00002030-199003000-00010 2350443

[18] Connor MD Lammie GA Bell JE Warlow CP Simmonds P Brettle RD. Cerebral infarction in adult AIDS patients: observations from the Edinburgh HIV autopsy cohort. Stroke. (2000) 31:2117–26. 10.1161/01.STR.31.9.2117 10978040

[19] Pinto AN. AIDS/HIV infection and cerebrovascular disease. In: Seminars in Cerebrovascular Diseases and Stroke. Vol. 5. WB Saunders (2005). p. 40–6. 10.1053/j.scds.2005.04.015

[20] Alonso A Barnes AE Guest JL Shah A Shao IY Marconi V. HIV infection and incidence of cardiovascular diseases: an analysis of a large healthcare database. J Am Heart Assoc. (2019) 8:e012241. 10.1161/JAHA.119.012241 31266386 PMC6662120

[21] Sico JJ Chang C-CH So-Armah K Justice AC Hylek E Skanderson M. HIV status and the risk of ischemic stroke among men. Neurology. (2015) 84:1933–40. 10.1212/WNL.0000000000001560 25862803 PMC4433456

[22] Durand M Sheehy O Baril JG LeLorier J Tremblay CL. Risk of spontaneous intracranial hemorrhage in HIV-infected individuals: a population-based cohort study. J Stroke Cerebrovasc Dis. (2013) 22:e34–41. 10.1016/j.jstrokecerebrovasdis.2012.03.014 22554568

[23] Chow FC He W Bacchetti P Regan S Feske SK Meigs JB. Elevated rates of intracerebral hemorrhage in individuals from a US clinical care HIV cohort. Neurology. (2014) 83:1705–11. 10.1212/WNL.0000000000000958 25280902 PMC4239837

[24] Rasmussen LD Engsig FN Christensen H Gerstoft J Kronborg G Obel NJA. Risk of cerebrovascular events in persons with and without HIV: a Danish nationwide population-based cohort study. AIDS. (2011) 25:1637–46. 10.1097/QAD.0b013e3283493fb0 21646903

[25] Fedele F Bruno N Mancone M. Cardiovascular risk factors and HIV disease. AIDS Rev. (2011) 13:119–29. 21587343

[26] Triant VA Perez J Regan S Massaro JM Meigs JB Grinspoon SK. Cardiovascular risk prediction functions underestimate risk in HIV infection. Circulation. (2018) 137:2203–14. 10.1161/CIRCULATIONAHA.117.028975 29444987 PMC6157923

[27] Deeks SG Phillips AN. HIV infection, antiretroviral treatment, ageing, and non-AIDS related morbidity. BMJ. (2009) 338:a3172. 10.1136/bmj.a3172 19171560

[28] Friis-Møller N Sabin CA Weber R d'Arminio Monforte A El-Sadr WM Reiss P. Combination antiretroviral therapy and the risk of myocardial infarction. N Engl J Med. (2003) 349:1993–2003. 10.1056/NEJMoa030218 14627784

[29] Benjamin LA Allain TJ Mzinganjira H Connor MD Smith C Lucas S. The role of human immunodeficiency virus–associated vasculopathy in the etiology of stroke. J Infect Dis. (2017) 216:545–53. 10.1093/infdis/jix340 28931222 PMC5853476

[30] Feinstein MJ Bogorodskaya M Bloomfield GS Vedanthan R Siedner MJ Kwan GF. Cardiovascular complications of HIV in endemic countries. Curr Cardiol Rep. (2016) 18:113. 10.1007/s11886-016-0794-x 27730474 PMC6717318

[31] Mlay M Bakari M. The prevalence of HIV among patients admitted with stroke at the Muhimbili national hospital, dar es salaam, Tanzania. Tanzan J Health Res. (2010) 12:105–13. 10.4314/thrb.v12i2.56397

[32] Grinspoon S Carr A. Cardiovascular risk and body-fat abnormalities in HIV-infected adults. N Engl J Med. (2005) 352:48–62. 10.1056/NEJMra041811 15635112

[33] Chow FC Regan S Feske S Meigs JB Grinspoon SK Triant VA. Comparison of ischemic stroke incidence in HIV-infected and non-HIV-infected patients in a US health care system. J Acquir Immune Defic Syndr. (2012) 60:351–8. 10.1097/QAI.0b013e31825c7f24 22580566 PMC3670086

[34] Barnes RP Lacson JC Bahrami H. hiv infection and risk of cardiovascular diseases beyond coronary artery disease. Curr Atheroscler Rep. (2017) 19:20. 10.1007/s11883-017-0652-3 28315199 PMC6066370

[35] Pereyra F Lo J Triant VA Wei J Buzon MJ Fitch KV. Increased coronary atherosclerosis and immune activation in HIV-1 elite controllers. AIDS. (2012) 26:2409–12. 10.1097/QAD.0b013e32835a9950 23032411 PMC3660105

[36] Phillips AN Carr A Neuhaus J Visnegarwala F Prineas R Burman WJ. Interruption of antiretroviral therapy and risk of cardiovascular disease in persons with HIV-1 infection: exploratory analyses from the SMART trial. Antivir Ther. (2008) 13:177–87. 18505169 10.1177/135965350801300215

[37] Hsue PY Hunt PW Schnell A Kalapus SC Hoh R Ganz P. Role of viral replication, antiretroviral therapy, and immunodeficiency in HIV-associated atherosclerosis. AIDS. (2009) 23:1059–67. 10.1097/QAD.0b013e32832b514b 19390417 PMC2691772

[38] Gutierrez J Glenn M Isaacson RS Marr AD Mash D Petito CJS. Thinning of the arterial media layer as a possible preclinical stage in HIV vasculopathy: a pilot study. Stroke. (2012) 43:1156–8. 10.1161/STROKEAHA.111.643387 22198982

[39] Gutierrez J Rosoklija G Murray J Chon C Elkind MS Goldman JE. A quantitative perspective to the study of brain arterial remodeling of donors with and without HIV in the brain arterial remodeling study (BARS). Front Physiol. (2014) 5:56. 10.3389/fphys.2014.00056 24600402 PMC3928551

[40] Gutierrez J Menshawy K Gonzalez M Goldman J Elkind MS Marshall R. Brain large artery inflammation associated with HIV and large artery remodeling. AIDS. (2016) 30:415–23. 10.1097/QAD.0000000000000927 26765935 PMC5203930

[41] Mateen FJ Post WS Sacktor N Abraham AG Becker JT Smith BR. Long-term predictive value of the Framingham risk score for stroke in HIV-positive vs HIV-negative men. Neurology. (2013) 81:2094–102. 10.1212/01.wnl.0000437296.97946.73 24212385 PMC3863354

[42] Benjamin L Khoo S. HIV infection and stroke. Handb Clin Neurol. (2018) 152:187–200. 10.1016/B978-0-444-63849-6.00015-3 29604976

[43] Cysique LA Brew BJ. Vascular cognitive impairment and HIV-associated neurocognitive disorder: a new paradigm. J Neurovirol. (2019) 25:710–21. 10.1007/s13365-018-0706-5 30635846

[44] Chow FC Wilson MR Kunling W Ellis RJ Bosch RJ Linas BPJA. Stroke incidence is highest in women and non-hispanic blacks living with HIV in the ALLRT cohort. AIDS. (2018) 32:1125. 10.1097/QAD.0000000000001799 29746317 PMC5955006

[45] Chow FC Lyass A Mahoney TF Massaro JM Triant VA Wu K. Baseline 10-year cardiovascular risk scores predict cognitive function in older persons, and particularly women, living with human immunodeficiency virus infection. Clin Infect Dis. (2020) 71:3079–85. 10.1093/cid/ciz1214 31899478 PMC7819524

[46] Montoya JL Iudicello J Oppenheim HA Fazeli PL Potter M Ma Q. Coagulation imbalance and neurocognitive functioning in older HIV-positive adults on suppressive antiretroviral therapy. AIDS. (2017) 31:787–95. 10.1097/QAD.0000000000001404 28099190 PMC5466004

[47] Hollestelle SC de Vries MR van Keulen JK Schoneveld AH Vink A Strijder CF. Toll-like receptor 4 is involved in outward arterial remodeling. Circulation. (2004) 109:393–8. 10.1161/01.CIR.0000109140.51366.72 14699006

[48] Mulvany MJ Baumbach GL Aalkjaer C Heagerty AM Korsgaard N Schiffrin EL. Vascular remodeling. Hypertension. (1996) 28:505–6. 8794840

[49] Taylor AJ Burke AP Farb A Yousefi P Malcom GT Smialek J. Arterial remodeling in the left coronary system: the role of high-density lipoprotein cholesterol. J Am Coll Cardiol. (1999) 34:760–7. 10.1016/S0735-1097(99)00275-2 10483958

[50] Schoenhagen P Ziada KM Vince DG Nissen SE Tuzcu EM. Arterial remodeling and coronary artery disease: the concept of “dilated” versus “obstructive” coronary atherosclerosis. J Am Coll Cardiol. (2001) 38:297–306. 10.1016/S0735-1097(01)01374-2 11499716

[51] Stein JH Klein MA Bellehumeur JL McBride PE Wiebe DA Otvos JD. Use of human immunodeficiency virus-1 protease inhibitors is associated with atherogenic lipoprotein changes and endothelial dysfunction. Circulation. (2001) 104:257–62. 10.1161/01.CIR.104.3.257 11457741

[52] Bonnet D Aggoun Y Szezepanski I Bellal N Blanche S. Arterial stiffness and endothelial dysfunction in HIV-infected children. AIDS. (2004) 18:1037–41. 10.1097/00002030-200404300-00012 15096807

[53] Dube MP Lipshultz SE Fichtenbaum CJ Greenberg R Schecter AD Fisher SD. Effects of HIV infection and antiretroviral therapy on the heart and vasculature. Circulation. (2008) 118:e36–40. 10.1161/CIRCULATIONAHA.107.189625 18566318

[54] Vos AG Idris NS Barth RE Klipstein-Grobusch K Grobbee DE. Pro-Inflammatory markers in relation to cardiovascular disease in HIV Infection. A systematic review. PLoS ONE. (2016) 11:e0147484. 10.1371/journal.pone.0147484 26808540 PMC4726827

[55] Nou E Lo J Grinspoon SK. Inflammation, immune activation, and cardiovascular disease in HIV. AIDS. (2016) 30:1495–509. 10.1097/QAD.0000000000001109 27058351 PMC4889507

[56] Zungsontiporn N Tello RR Zhang G Mitchell BI Budoff M Kallianpur KJ. Non-classical monocytes and monocyte chemoattractant protein-1 (MCP-1) correlate with coronary artery calcium progression in chronically hiv-1 infected adults on stable antiretroviral therapy. PLoS ONE. (2016) 11:e0149143. 10.1371/journal.pone.0149143 26867220 PMC4750941

[57] Gutierrez J Goldman J Dwork AJ Elkind MS Marshall RS Morgello S. Brain arterial remodeling contribution to nonembolic brain infarcts in patients with HIV. Neurology. (2015) 85:1139–45. 10.1212/WNL.0000000000001976 26320196 PMC4603890

[58] Re MC Furlini G Cenacchi G Preda P La Placa M. Human immunodeficiency virus type 1 infection of endothelial cells *in vitro*. Microbiologica. (1991) 14:149–52. 1713288

[59] Chi D Henry J Kelley J Thorpe R Smith JK Krishnaswamy G. The effects of HIV infection on endothelial function. Endothelium. (2000) 7:223–42. 10.3109/10623320009072210 11201521

[60] Kuller LH Tracy R Belloso W De Wit S Drummond F Lane HC. Inflammatory and coagulation biomarkers and mortality in patients with HIV infection. PLoS Med. (2008) 5:e203. 10.1371/journal.pmed.0050203 18942885 PMC2570418

[61] Burdo TH Lentz MR Autissier P Krishnan A Halpern E Letendre S. Soluble CD163 made by monocyte/macrophages is a novel marker of HIV activity in early and chronic infection prior to and after anti-retroviral therapy. J Infect Dis. (2011) 204:154–63. 10.1093/infdis/jir214 21628670 PMC3105035

[62] Hanna DB Lin J Post WS Hodis HN Xue X Anastos K. Association of macrophage inflammation biomarkers with progression of subclinical carotid artery atherosclerosis in HIV-infected women and men. J Infect Dis. (2017) 215:1352–61. 10.1093/infdis/jix082 28199691 PMC5722037

[63] Paladugu R Fu W Conklin BS Lin PH Lumsden AB Yao Q. Hiv tat protein causes endothelial dysfunction in porcine coronary arteries. J Vasc Surg. (2003) 38:549–55; discussion 55–6. 10.1016/S0741-5214(03)00770-5 12947275

[64] Kim TA Avraham HK Koh YH Jiang S Park IW Avraham S. HIV-1 Tat-mediated apoptosis in human brain microvascular endothelial cells. J Immunol. (2003) 170:2629–37. 10.4049/jimmunol.170.5.2629 12594291

[65] Marecki J Cool C Voelkel N Luciw P Flores S. Evidence for vascular remodeling in the lungs of macaques infected with simian immunodeficiency virus/HIV NEF recombinant virus. Chest. (2005) 128 (6 Suppl):621S−2S. 10.1378/chest.128.6_suppl.621S 16373878

[66] Duffy P Wang X Lin PH Yao Q Chen C. HIV Nef protein causes endothelial dysfunction in porcine pulmonary arteries and human pulmonary artery endothelial cells. J Surg Res. (2009) 156:257–64. 10.1016/j.jss.2009.02.005 19540523 PMC2760402

[67] Anand AR Rachel G Parthasarathy D. HIV proteins and endothelial dysfunction: implications in cardiovascular disease. Front Cardiovasc Med. (2018) 5:185. 10.3389/fcvm.2018.00185 30619892 PMC6305718

[68] De Alwis PM Smith B Wu T Artrip C Steinbach S Morse C. *In-vivo* MRI reveals changes to intracerebral vasculature caliber in HIV infection. Front Neurol. (2019) 10:687. 10.3389/fneur.2019.00687 31297086 PMC6607694

[69] Anand AR Ganju RK. HIV-1 gp120-mediated apoptosis of T cells is regulated by the membrane tyrosine phosphatase CD45. J Biol Chem. (2006) 281:12289–99. 10.1074/jbc.M511786200 16524887

[70] Anand AR Prasad A Bradley RR Deol YS Nagaraja T Ren X. HIV-1 gp120-induced migration of dendritic cells is regulated by a novel kinase cascade involving Pyk2, p38 MAP kinase, and LSP1. Blood. (2009) 114:3588–600. 10.1182/blood-2009-02-206342 19700666 PMC2766677

[71] Munshi N Balasubramanian A Koziel M Ganju RK Groopman JE. Hepatitis C and human immunodeficiency virus envelope proteins cooperatively induce hepatocytic apoptosis via an innocent bystander mechanism. J Infect Dis. (2003) 188:1192–204. 10.1086/378643 14551890

[72] Freedman BD Liu QH Del Corno M Collman RG. HIV-1 gp120 chemokine receptor-mediated signaling in human macrophages. Immunol Res. (2003) 27:261–76. 10.1385/IR:27:2-3:261 12857973

[73] Banda NK Bernier J Kurahara DK Kurrle R Haigwood N Sekaly RP. Crosslinking CD4 by human immunodeficiency virus gp120 primes T cells for activation-induced apoptosis. J Exp Med. (1992) 176:1099–106. 10.1084/jem.176.4.1099 1402655 PMC2119378

[74] Hijmans JG Stockleman K Reiakvam W Levy MaV Brewster LM Bammert TD. Effects of HIV-1 gp120 and tat on endothelial cell sensescence and senescence-associated micro RNA s. Physiol Rep. (2018) 6:e13647. 10.14814/phy2.13647 29595877 PMC5875545

[75] Katsuumi G Shimizu I Yoshida Y Minamino T. Vascular senescence in cardiovascular and metabolic diseases. Front Cardiovasc Med. (2018) 5:18. 10.3389/fcvm.2018.00018 29556500 PMC5845435

[76] Bavinger C Bendavid E Niehaus K Olshen RA Olkin I Sundaram V. Risk of cardiovascular disease from antiretroviral therapy for HIV: a systematic review. PLoS One. (2013) 8:e59551. 10.1371/journal.pone.0059551 23555704 PMC3608726

[77] Ene L Duiculescu D Ruta SM. How much do antiretroviral drugs penetrate into the central nervous system? J Med Life. (2011) 4:432–9. 22514580 PMC3227164

[78] Mukerji SS Misra V Lorenz DR Uno H Morgello S Franklin D. Impact of antiretroviral regimens on cerebrospinal fluid viral escape in a prospective multicohort study of antiretroviral therapy-experienced human immunodeficiency virus-1–infected adults in the United States. Clin Infect Dis. (2018) 67:1182–90. 10.1093/cid/ciy267 29617912 PMC6160603

[79] Pérez-Valero I Ellis R Heaton R Deutsch R Franklin D Clifford DB. Cerebrospinal fluid viral escape in aviremic HIV-infected patients receiving antiretroviral therapy: prevalence, risk factors and neurocognitive effects. AIDS. (2019) 33:475–81. 10.1097/QAD.0000000000002074 30702516 PMC6361539

[80] Warriner AH Burkholder GA Overton ET. HIV-related metabolic comorbidities in the current ART era. Infect Dis Clin North Am. (2014) 28:457–76. 10.1016/j.idc.2014.05.003 25151566

[81] Islam F Wu J Jansson J Wilson DP. Relative risk of cardiovascular disease among people living with HIV: a systematic review and meta-analysis. HIV Med. (2012) 13:453–68. 10.1111/j.1468-1293.2012.00996.x 22413967

[82] Hanna DB Post WS Deal JA Hodis HN Jacobson LP Mack WJ. HIV infection is associated with progression of subclinical carotid atherosclerosis. Clin Infect Dis. (2015) 61:640–50. 10.1093/cid/civ325 25904369 PMC4607734

[83] Hsue PY Lo JC Franklin A Bolger AF Martin JN Deeks SG. Progression of atherosclerosis as assessed by carotid intima-media thickness in patients with HIV infection. Circulation. (2004) 109:1603–8. 10.1161/01.CIR.0000124480.32233.8A 15023877

[84] Patel K Wang J Jacobson DL Lipshultz SE Landy DC Geffner ME. Aggregate risk of cardiovascular disease among adolescents perinatally infected with the human immunodeficiency virus. Circulation. (2014) 129:1204–12. 10.1161/CIRCULATIONAHA.113.001978 24366631 PMC3991841

[85] Shenoy A Dwork A Elkind MSV Marshall R Morgello S Gutierrez J. Brain large artery lymphocytic inflammation and human immunodeficiency virus-related brain arterial remodeling. J Virol. (2018) 92e0081–18. 10.1128/JVI.00081-18 29618649 PMC5974492

[86] Gherardi R Belec L Mhiri C Gray F Lescs MC Sobel A. The spectrum of vasculitis in human immunodeficiency virus–infected patients. A clinicopathologic evaluation. Arthritis Rheum. (1993) 36:1164–74. 10.1002/art.1780360818 8343192

[87] Chetty R. Vasculitides associated with HIV infection. J Clin Pathol. (2001) 54:275–8. 10.1136/jcp.54.4.275 11304843 PMC1731393

[88] Kearns AC Liu F Dai S Robinson JA Kiernan E Tesfaye Cheru L. Caspase-1 activation is related with HIV-associated atherosclerosis in an HIV transgenic mouse model and HIV patient cohort. Arterioscler Thromb Vasc Biol. (2019) 39:1762–75. 10.1161/ATVBAHA.119.312603 31315440 PMC6703939

[89] Gutierrez J Hatleberg CI Evans H Yin MT. Role of pre-stroke immunity in ischemic stroke mechanism among patients with HIV. AIDS Care. (2019) 31:270–4. 10.1080/09540121.2018.1510096 30126294 PMC6289722

[90] Wasserman BA. Advanced contrast-enhanced MRI for looking beyond the lumen to predict stroke: building a risk profile for carotid plaque. Stroke. (2010) 41 (10 Suppl):S12–6. 10.1161/STROKEAHA.110.596288 20876485

[91] Mee TC Aepfelbacher J Krakora R Chairez C Kvaratskhelia N Smith B. Carotid magnetic resonance imaging in persons living with HIV and 10-year atherosclerotic cardiovascular disease risk score. Antivir Ther. (2018) 23:695–8. 10.3851/IMP3258 30088806

[92] Rose KA Vera JH Drivas P Banya W Keenan N Pennell DJ. Atherosclerosis is evident in treated HIV-infected subjects with low cardiovascular risk by carotid cardiovascular magnetic resonance. J Acquir Immune Defic Syndr. (2016) 71:514–21. 10.1097/QAI.0000000000000900 26579986 PMC4782218

[93] Schoepf IC Buechel RR Kovari H Hammoud DA Tarr PE. Subclinical atherosclerosis imaging in people living with HIV. J Clin Med. (2019) 8:1125. 10.3390/jcm8081125 31362391 PMC6723163

[94] LaBounty TM Hardy WD Fan Z Yumul R Li D Dharmakumar R. Carotid artery thickness is associated with chronic use of highly active antiretroviral therapy in patients infected with human immunodeficiency virus: a 3.0 Tesla magnetic resonance imaging study. HIV Med. (2016) 17:516–23. 10.1111/hiv.12351 26634886 PMC5477634

[95] Ward MR Pasterkamp G Yeung AC Borst C. Arterial remodeling. Mechanisms and clinical implications. Circulation. (2000) 102:1186–91. 10.1161/01.CIR.102.10.1186 10973850

[96] Gutierrez J Menshawy K Goldman J Dwork AJ Elkind MS Marshall RS. Metalloproteinases and brain arterial remodeling among individuals with and those without HIV infection. J Infect Dis. (2016) 214:1329–35. 10.1093/infdis/jiw385 27549585 PMC5079372

[97] He R Guo DC Estrera AL Safi HJ Huynh TT Yin Z. Characterization of the inflammatory and apoptotic cells in the aortas of patients with ascending thoracic aortic aneurysms and dissections. J Thorac Cardiovasc Surg. (2006) 131:671–8. 10.1016/j.jtcvs.2005.09.018 16515922

[98] Newman KM Jean-Claude J Li H Scholes JV Ogata Y Nagase H. Cellular localization of matrix metalloproteinases in the abdominal aortic aneurysm wall. J Vasc Surg. (1994) 20:814–20. 10.1016/S0741-5214(94)70169-5 7526009

[99] Dogan A Tuzun N Turker Y Akcay S Kaya S Ozaydin M. Matrix metalloproteinases and inflammatory markers in coronary artery ectasia: their relationship to severity of coronary artery ectasia. Coron Artery Dis. (2008) 19:559–63. 10.1097/MCA.0b013e3283109079 19005290

[100] Ortiz G Koch S Romano JG Forteza AM Rabinstein AA. Mechanisms of ischemic stroke in HIV-infected patients. Neurology. (2007) 68:1257–61. 10.1212/01.wnl.0000259515.45579.1e 17438215

[101] Mochan A Modi M Modi G. Stroke in black South African HIV-positive patients: a prospective analysis. Stroke. (2003) 34:10–5. 10.1161/01.STR.0000043821.35051.FA 12511743

[102] Guedes BF Gomes HR Lucato LT Puglia P Jr Nitrini R Castro LH. Human immunodeficiency virus-associated vasculopathy with CNS compartmentalization of HIV-1. J Neurovirol. (2015) 21:101–4. 10.1007/s13365-014-0307-x 25537635

[103] Lantos PL McLaughlin JE Schoitz CL Berry CL Tighe JR. Neuropathology of the brain in HIV infection. Lancet. (1989) 1:309–11. 10.1016/S0140-6736(89)91316-0 2563464

[104] Oliviero U Bonadies G Apuzzi V Foggia M Bosso G Nappa S. Human immunodeficiency virus per se exerts atherogenic effects. Atherosclerosis. (2009) 204:586–9. 10.1016/j.atherosclerosis.2008.10.012 19084229

[105] Solages A Vita JA Thornton DJ Murray J Heeren T Craven DE. Endothelial function in HIV-infected persons. Clin Infect Dis. (2006) 42:1325–32. 10.1086/503261 16586393 PMC2737346

[106] Seaberg EC Benning L Sharrett AR Lazar JM Hodis HN Mack WJ. Association between human immunodeficiency virus infection and stiffness of the common carotid artery. Stroke. (2010) 41:2163–70. 10.1161/STROKEAHA.110.583856 20798374 PMC2972735

[107] Burke AP Kolodgie FD Farb A Weber D Virmani R. Morphological predictors of arterial remodeling in coronary atherosclerosis. Circulation. (2002) 105:297–303. 10.1161/hc0302.102610 11804983

[108] Anderson TJ. Arterial stiffness or endothelial dysfunction as a surrogate marker of vascular risk. Can J Cardiol. (2006) 22:72B−80B. 10.1016/S0828-282X(06)70990-4 16498516 PMC2780833

[109] Saylor D Dickens AM Sacktor N Haughey N Slusher B Pletnikov M. HIV-associated neurocognitive disorder—pathogenesis and prospects for treatment. Nat Rev Neurol. (2016) 12:234–48. 10.1038/nrneurol.2016.27 26965674 PMC4937456

[110] Gannon P Khan MZ Kolson DL. Current understanding of HIV-associated neurocognitive disorders pathogenesis. Curr Opin Neurol. (2011) 24:275–83. 10.1097/WCO.0b013e32834695fb 21467932 PMC3683661

[111] Montoya JL Iudicello J Fazeli PL Hong S Potter M Ellis RJ. Elevated markers of vascular remodeling and arterial stiffness are associated with neurocognitive function in older HIV+ adults on suppressive antiretroviral therapy. J Acquir Immune Defic Syndr. (2017) 74:134–41. 10.1097/QAI.0000000000001230 27828873 PMC5233588

[112] Gelman BB. Neuropathology of HAND with suppressive antiretroviral therapy: encephalitis and neurodegeneration reconsidered. Curr HIV/AIDS Rep. (2015) 12:272–9. 10.1007/s11904-015-0266-8 25860316 PMC4427627

[113] Levine AJ Soontornniyomkij V Masliah E Sinsheimer JS Ji SS Horvath S. A candidate gene study of intermediate histopathological phenotypes in HIV-associated neurocognitive disorders. J Neurovirol. (2020) 26:509–10. 10.1007/s13365-020-00871-y 32632672

[114] Clifford DB Ances BM. HIV-associated neurocognitive disorder. Lancet Infect Dis. (2013) 13:976–86. 10.1016/S1473-3099(13)70269-X 24156898 PMC4108270

[115] Vago L Bonetto S Nebuloni M Duca P Carsana L Zerbi P. Pathological findings in the central nervous system of AIDS patients on assumed antiretroviral therapeutic regimens: retrospective study of 1597 autopsies. AIDS. (2002) 16:1925–8. 10.1097/00002030-200209270-00009 12351952

[116] Bandera A Taramasso L Bozzi G Muscatello A Robinson JA Burdo TH. HIV-associated neurocognitive impairment in the modern ART era: are we close to discovering reliable biomarkers in the setting of virological suppression? Front Aging Neurosci. (2019) 11:187. 10.3389/fnagi.2019.00187 31427955 PMC6687760

[117] Peluso MJ Meyerhoff DJ Price RW Peterson J Lee E Young AC. Cerebrospinal fluid and neuroimaging biomarker abnormalities suggest early neurological injury in a subset of individuals during primary HIV infection. J Infect Dis. (2013) 207:1703–12. 10.1093/infdis/jit088 23460748 PMC3636785

[118] Nguyen TP Soukup VM Gelman BB. Persistent hijacking of brain proteasomes in HIV-associated dementia. Am J Pathol. (2010) 176:893–902. 10.2353/ajpath.2010.090390 20035054 PMC2808094

[119] Everall IP Hansen LA Masliah E. The shifting patterns of HIV encephalitis neuropathology. Neurotox Res. (2005) 8:51–61. 10.1007/BF03033819 16260385

[120] Mackiewicz MM Overk C Achim CL Masliah E. Pathogenesis of age-related HIV neurodegeneration. J Neurovirol. (2019) 25:622–33. 10.1007/s13365-019-00728-z 30790184 PMC6703984

[121] Fields J Dumaop W Langford TD Rockenstein E Masliah E. Role of neurotrophic factor alterations in the neurodegenerative process in HIV associated neurocognitive disorders. J Neuroimmune Pharmacol. (2014) 9:102–16. 10.1007/s11481-013-9520-2 24510686 PMC3973421

[122] Milanini B Valcour V. Differentiating HIV-associated neurocognitive disorders from Alzheimer's disease: an emerging issue in geriatric NeuroHIV. Curr HIV/AIDS Rep. (2017) 14:123–32. 10.1007/s11904-017-0361-0 28779301 PMC5823609

[123] Smail RC Brew BJ. HIV-associated neurocognitive disorder. Handb Clin Neurol. (2018) 152:75–97. 10.1016/B978-0-444-63849-6.00007-4 29604986

[124] Heaton R Franklin D Letendre S Ellis R Fennema-Notestine C Vaida F. Aging amplifies HIV neurocognitive impairment: the effects may be related to vascular and metabolic factors. J Neurovirol. (2012) 18:S46.

[125] Peterson TE Huppler Hullsiek K Wyman Engen N Kumarasamy N Lebech AM Liappis A. Inflammation associates with impaired small arterial elasticity early in HIV disease. Open Forum Infect Dis. (2018) 5:ofy117. 10.1093/ofid/ofy117 29942822 PMC6007791

[126] Subramanian S Tawakol A Burdo TH Abbara S Wei J Vijayakumar J. Arterial inflammation in patients with HIV. JAMA. (2012) 308:379–86. 10.1001/jama.2012.6698 22820791 PMC3724172

[127] Longenecker CT Jiang Y Yun CH Debanne S Funderburg NT Lederman MM. Perivascular fat, inflammation, and cardiovascular risk in HIV-infected patients on antiretroviral therapy. Int J Cardiol. (2013) 168:4039–45. 10.1016/j.ijcard.2013.06.059 23886531 PMC3805774

[128] Benjamin LA Bryer A Lucas S Stanley A Allain TJ Joekes E. Arterial ischemic stroke in HIV: defining and classifying etiology for research studies. Neurol Neuroimmunol Neuroinflamm. (2016) 3:e254. 10.1212/NXI.0000000000000254 27386505 PMC4929887

[129] Moulignier A Savatovsky J Assoumou L Lescure FX Lamirel C Godin O. Silent cerebral small-vessel disease is twice as prevalent in middle-aged individuals with well-controlled, combination antiretroviral therapy–treated human immunodeficiency virus (HIV) than in HIV-uninfected individuals. Clin Infect Dis. (2018) 66:1762–9. 10.1093/cid/cix1075 29244126

[130] Lui G Ma RC Chook P Wong CK Tam CH Chan MH. Brief report: progression of atherosclerosis in HIV-infected individuals—prospective data from an asian cohort. JAIDS. (2017) 75:198–202. 10.1097/QAI.0000000000001358 28498145

[131] Pantoni L. Cerebral small vessel disease: from pathogenesis and clinical characteristics to therapeutic challenges. Lancet Neurol. (2010) 9:689–701. 10.1016/S1474-4422(10)70104-6 20610345

[132] Sanford R Strain J Dadar M Maranzano J Bonnet A Mayo NE. HIV infection and cerebral small vessel disease are independently associated with brain atrophy and cognitive impairment. AIDS. (2019) 33:1197–205. 10.1097/QAD.0000000000002193 30870193 PMC7924885

[133] Soontornniyomkij V Umlauf A Chung SA Cochran ML Soontornniyomkij B Gouaux B. HIV protease inhibitor exposure predicts cerebral small vessel disease. AIDS. (2014) 28:1297–306. 10.1097/QAD.0000000000000262 24637542 PMC4071161

[134] Bertrand L Meroth F Tournebize M Leda AR Sun E Toborek M. Targeting the HIV-infected brain to improve ischemic stroke outcome. Nat Commun. (2019) 10:2009. 10.1038/s41467-019-10046-x 31043599 PMC6494822

[135] Fazeli PL Crowe M Ross LA Wadley V Ball K Vance DE. Cognitive functioning in adults aging with HIV: a cross-sectional analysis of cognitive subtypes and influential factors. J Clin Res HIV AIDS Prev. (2014) 1:155–69. 10.14302/issn.2324-7339.jcrhap-13-191 25386565 PMC4224145

[136] Valcour V Shikuma C Shiramizu B Watters M Poff P Selnes O. Higher frequency of dementia in older HIV-1 individuals: the hawaii aging with HIV-1 cohort. Neurology. (2004) 63:822–7. 10.1212/01.WNL.0000134665.58343.8D 15365130 PMC1382180

[137] Joska JA Westgarth-Taylor J Myer L Hoare J Thomas KG Combrinck M. Characterization of HIV-associated neurocognitive disorders among individuals starting antiretroviral therapy in South Africa. AIDS Behav. (2011) 15:1197–203. 10.1007/s10461-010-9744-6 20614176

[138] Joska JA Westgarth-Taylor J Hoare J Thomas KG Paul R Myer L. Neuropsychological outcomes in adults commencing highly active anti-retroviral treatment in South Africa: a prospective study. BMC Infect Dis. (2012) 12:39. 10.1186/1471-2334-12-39 22335937 PMC3356227

[139] Marquine MJ Umlauf A Rooney A Fazeli PL Gouaux B Woods SP. The veterans aging cohort study (VACS) Index is associated with concurrent risk for neurocognitive impairment. J Acquir Immune Defic Syndr. (2014) 65:190–7. 10.1097/QAI.0000000000000008 24442225 PMC3907119

[140] Wright EJ Grund B Cysique LA Robertson K Brew BJ Collins G. Factors associated with neurocognitive test performance at baseline: a substudy of the INSIGHT strategic timing of antiretroviral treatment (START) trial. HIV Med. (2015) 16:97–108. 10.1111/hiv.12238 25711328

[141] Heaton RK Clifford DB Franklin DR Jr Woods SP Ake C Vaida F. HIV-associated neurocognitive disorders persist in the era of potent antiretroviral therapy: CHARTER study. Neurology. (2010) 75:2087–96. 10.1212/WNL.0b013e318200d727 21135382 PMC2995535

[142] Sacktor N Skolasky RL Seaberg E Munro C Becker JT Martin E. Prevalence of HIV-associated neurocognitive disorders in the multicenter AIDS cohort study. Neurology. (2016) 86:334–40. 10.1212/WNL.0000000000002277 26718568 PMC4776086

[143] Gutierrez J Byrd D Yin MT Morgello S. Relationship between brain arterial pathology and neurocognitive performance among individuals with human immunodeficiency virus. Clin Infect Dis. (2019) 68:490–7. 10.1093/cid/ciy501 30107467 PMC6336905

[144] Wright EJ Grund B Robertson K Brew BJ Roediger M Bain MP. Cardiovascular risk factors associated with lower baseline cognitive performance in HIV-positive persons. Neurology. (2010) 75:864–73. 10.1212/WNL.0b013e3181f11bd8 20702792 PMC2938971

[145] Gutierrez J Honig L Elkind MS Mohr JP Goldman J Dwork AJ. Brain arterial aging and its relationship to alzheimer dementia. Neurology. (2016) 86:1507–15. 10.1212/WNL.0000000000002590 26984942 PMC4836884

[146] Rincon F Sacco RL Kranwinkel G Xu Q Paik MC Boden-Albala B. Incidence and risk factors of intracranial atherosclerotic stroke: the Northern Manhattan stroke study. Cerebrovasc Dis. (2009) 28:65–71. 10.1159/000219299 19468217 PMC2914420

[147] Ohira T Shahar E Chambless LE Rosamond WD Mosley TH Jr Folsom AR. Risk factors for ischemic stroke subtypes: the atherosclerosis risk in communities study. Stroke. (2006) 37:2493–8. 10.1161/01.STR.0000239694.19359.88 16931783

[148] Gutierrez J Elkind MS Virmani R Goldman J Honig L Morgello S. A pathological perspective on the natural history of cerebral atherosclerosis. Int J Stroke. (2015) 10:1074–80. 10.1111/ijs.12496 25854637 PMC4583838

[149] Valcour VG Shikuma CM Shiramizu BT Williams AE Watters MR Poff PW. Diabetes, insulin resistance, and dementia among HIV-1–infected patients. J Acquir Immune Defic Syndr. (2005) 38:31. 10.1097/00126334-200501010-00006 15608521 PMC1415271

[150] McCutchan JA Marquie-Beck JA Fitzsimons CA Letendre SL Ellis RJ Heaton RK. Role of obesity, metabolic variables, and diabetes in HIV-associated neurocognitive disorder. Neurology. (2012) 78:485–92. 10.1212/WNL.0b013e3182478d64 22330412 PMC3280051

[151] Kamat A Lyons JL Misra V Uno H Morgello S Singer EJ. Monocyte activation markers in cerebrospinal fluid associated with impaired neurocognitive testing in advanced HIV infection. J Acquir Immune Defic Syndr. (2012) 60:234–43. 10.1097/QAI.0b013e318256f3bc 22569268 PMC3383928

[152] Blokhuis C Mutsaerts H Cohen S Scherpbier HJ Caan MWA Majoie C. Higher subcortical and white matter cerebral blood flow in perinatally HIV-infected children. Medicine. (2017) 96:e5891. 10.1097/MD.0000000000005891 28207506 PMC5319495

[153] Su T Mutsaerts HJ Caan MW Wit FW Schouten J Geurtsen GJ. Cerebral blood flow and cognitive function in HIV-infected men with sustained suppressed viremia on combination antiretroviral therapy. AIDS. (2017) 31:847–56. 10.1097/QAD.0000000000001414 28121708

[154] O'Brien JT Erkinjuntti T Reisberg B Roman G Sawada T Pantoni L. Vascular cognitive impairment. Lancet Neurol. (2003) 2:89–98. 10.1016/S1474-4422(03)00305-3 12849265

[155] Gutierrez J Albuquerque ALA Falzon L. HIV infection as vascular risk: a systematic review of the literature and meta-analysis. HIV infection as vascular risk: a systematic review of the literature and meta-analysis. PloS ONE. (2017) 12:e0176686. 10.1371/journal.pone.0176686 28493892 PMC5426615

[156] Lacson JC Barnes RP Bahrami H. Coronary artery disease in HIV-infected patients: downside of living longer. Curr Atheroscler Rep. (2017) 19:18. 10.1007/s11883-017-0651-4 28265887 PMC6066371

[157] Holloway CJ Boccara F. HIV-related cardiovascular disease: closing the gap in mortality. Curr Opin HIV AIDS. (2017) 12:509–12. 10.1097/COH.0000000000000420 28984701

[158] Hsue PY Deeks SG Hunt PW. Immunologic basis of cardiovascular disease in HIV-infected adults. J Infect Dis. (2012) 205 (Suppl. 3):S375–82. 10.1093/infdis/jis200 22577211 PMC3349295

[159] Elicer IM Byrd D Clark US Morgello S Robinson-Papp J. Motor function declines over time in human immunodeficiency virus and is associated with cerebrovascular disease, while HIV-associated neurocognitive disorder remains stable. J Neurovirol. (2018). 24:514–22. 10.1007/s13365-018-0640-6 29696578 PMC6309169

[160] Gelman BB Chen T Lisinicchia JG Soukup VM Carmical JR Starkey JM. The national NeuroAIDS tissue consortium brain gene array: two types of HIV-associated neurocognitive impairment. PLoS ONE. (2012) 7:e46178. 10.1371/journal.pone.0046178 23049970 PMC3458860

[161] Kallianpur AR Gittleman H Letendre S Ellis R Barnholtz-Sloan JS Bush WS. Cerebrospinal fluid ceruloplasmin, haptoglobin, and vascular endothelial growth factor are associated with neurocognitive impairment in adults with HIV infection. Mol Neurobiol. (2019) 56:3808–18. 10.1007/s12035-018-1329-9 30209774 PMC6952215

[162] Cysique LA Moffat K Moore DM Lane TA Davies NW Carr A. HIV, vascular and aging injuries in the brain of clinically stable HIV-infected adults: a (1)H MRS study. PLoS ONE. (2013) 8:e61738. 10.1371/journal.pone.0061738 23620788 PMC3631163

[163] Harezlak J Buchthal S Taylor M Schifitto G Zhong J Daar E. Persistence of HIV-associated cognitive impairment, inflammation, and neuronal injury in era of highly active antiretroviral treatment. AIDS. (2011) 25:625–33. 10.1097/QAD.0b013e3283427da7 21297425 PMC4326227

[164] Ulfhammer G Edén A Mellgren Å Fuchs D Zetterberg H Hagberg L. Persistent central nervous system immune activation following more than 10 years of effective HIV antiretroviral treatment. AIDS. (2018) 32:2171–8. 10.1097/QAD.0000000000001950 30005007

[165] Saloner R Cysique LA. HIV-Associated neurocognitive disorders: a global perspective. J Int Neuropsychol Soc. (2017) 23:860–9. 10.1017/S1355617717001102 29198283 PMC5939823

[166] Zhou P Yang XL Wang XG Hu B Zhang L Zhang W. A pneumonia outbreak associated with a new coronavirus of probable bat origin. Nature. (2020) 579:270–3. 10.1038/s41586-020-2012-7 32015507 PMC7095418

[167] Hoffmann M Kleine-Weber H Schroeder S Krüger N Herrler T Erichsen S. SARS-CoV-2 cell entry depends on ACE2 and TMPRSS2 and is blocked by a clinically proven protease inhibitor. Cell. (2020) 181:271–80. e8. 10.1016/j.cell.2020.02.052 32142651 PMC7102627

[168] Doobay MF Talman LS Obr TD Tian X Davisson RL Lazartigues E. Differential expression of neuronal ACE2 in transgenic mice with overexpression of the brain renin-angiotensin system. Am J Physiol Regul Integr Comp Physiol. (2007) 292:R373–81. 10.1152/ajpregu.00292.2006 16946085 PMC1761128

[169] Baig AM. Neurological manifestations in COVID-19 caused by SARS-CoV-2. CNS Neurosci Ther. (2020) 26:499–501. 10.1111/cns.13372 32266761 PMC7163592

[170] Chen J Xiao X Chen S Zhang C Chen J Yi D. Angiotensin-converting enzyme 2 priming enhances the function of endothelial progenitor cells and their therapeutic efficacy. Hypertension. (2013) 61:681–9. 10.1161/HYPERTENSIONAHA.111.00202 23266545 PMC4011714

[171] Chen J Zhao Y Chen S Wang J Xiao X Ma X. Neuronal over-expression of ACE2 protects brain from ischemia-induced damage. Neuropharmacology. (2014) 79:550–8. 10.1016/j.neuropharm.2014.01.004 24440367 PMC3992949

[172] Hess DC Eldahshan W Rutkowski E. COVID-19-Related stroke. Transl Stroke Res. (2020) 11:322–5. 10.1007/s12975-020-00818-9 32378030 PMC7202903

[173] Pugin D Vargas MI Thieffry C Schibler M Grosgurin O Pugin J. COVID-19–related encephalopathy responsive to high-dose glucocorticoids. Neurology. (2020) 95:543–6. 10.1212/WNL.0000000000010354 32680950

[174] Kuba K Imai Y Penninger JM. Multiple functions of angiotensin-converting enzyme 2 and its relevance in cardiovascular diseases. Circ J. (2013) 77:301–8. 10.1253/circj.CJ-12-1544 23328447

[175] Dubois C Panicot-Dubois L Gainor JF Furie BC Furie B. Thrombin-initiated platelet activation *in vivo* is vWF independent during thrombus formation in a laser injury model. J Clin Invest. (2007) 117:953–60. 10.1172/JCI30537 17380206 PMC1821068

[176] Atkinson BT Jasuja R Chen VM Nandivada P Furie B Furie BC. Laser-induced endothelial cell activation supports fibrin formation. Blood. (2010) 116:4675–83. 10.1182/blood-2010-05-283986 20675401 PMC2996123

[177] Sigel K Swartz T Golden E Paranjpe I Somani S Richter F. Coronavirus 2019 and people living with human immunodeficiency virus: outcomes for hospitalized patients in New York City. Clin Infect Dis. (2020) 71:2933–8. 10.1093/cid/ciaa880 32594164 PMC7337691

[178] Karmen-Tuohy S Carlucci PM Zervou FN Zacharioudakis IM Rebick G Klein E. Outcomes among HIV-positive patients hospitalized with COVID-19. J Acquir Immune Defic Syndr. (2020) 85:6–10. 10.1101/2020.05.07.20094797 32568770 PMC7446982

[179] Vizcarra P Perez-Elias MJ Quereda C Moreno A Vivancos MJ Dronda F. Description of COVID-19 in HIV-infected individuals: a single-centre, prospective cohort. Lancet HIV. (2020) 7:e554–64. 10.1016/S2352-3018(20)30164-8 32473657 PMC7255735

[180] del Amo J Polo R Moreno S Diaz A Martinez E. Incidence and severity of COVID-19 in HIV-positive persons receiving antiretroviral therapy. Ann Intern Med. (2020) 173:536–41. 10.7326/M20-3689 32589451 PMC7394316

[181] Johnson W Onuma O Owolabi M Sachdev S. Stroke: a global response is needed. Bull World Health Organ. (2016) 94:634. 10.2471/BLT.16.181636 27708464 PMC5034645

[182] Ortblad KF Lozano R Murray CJ. The burden of HIV: insights from the global burden of disease study 2010. AIDS. (2013) 27:2003–17. 10.1097/QAD.0b013e328362ba67 23660576 PMC3748855

[183] Abdallah A Chang JL O'Carroll CB Musubire A Chow FC Wilson AL. Stroke in human immunodeficiency virus-infected individuals in Sub-Saharan Africa (SSA): a systematic review. J Stroke Cerebrovasc Dis. (2018) 27:1828–36. 10.1016/j.jstrokecerebrovasdis.2018.02.016 29628338 PMC6641537

[184] Benjamin LA Corbett EL Connor MD Mzinganjira H Kampondeni S Choko A. HIV, antiretroviral treatment, hypertension, and stroke in Malawian adults: a case-control study. Neurology. (2016) 86:324–33. 10.1212/WNL.0000000000002278 26683649 PMC4776088

[185] Eshtehardi P McDaniel MC Dhawan SS Binongo JN Krishnan SK Golub L. Effect of intensive atorvastatin therapy on coronary atherosclerosis progression, composition, arterial remodeling, and microvascular function. J Invasive Cardiol. (2012) 24:522–9. 23043036

[186] Tunick PA Nayar AC Goodkin GM Mirchandani S Francescone S Rosenzweig BP. Effect of treatment on the incidence of stroke and other emboli in 519 patients with severe thoracic aortic plaque. Am J Cardiol. (2002) 90:1320–5. 10.1016/S0002-9149(02)02870-9 12480041

